# Dietary oxidized beef protein alters gut microbiota and induces colonic inflammatory damage in C57BL/6 mice

**DOI:** 10.3389/fnut.2022.980204

**Published:** 2022-09-02

**Authors:** Yantao Yin, Jiaming Cai, Lei Zhou, Lujuan Xing, Wangang Zhang

**Affiliations:** Key Laboratory of Meat Processing and Quality Control, Ministry of Education China, College of Food Science and Technology, Nanjing Agricultural University, Nanjing, China

**Keywords:** beef, protein oxidation, gut microbiota (GM), intestinal barrier, colon inflammation

## Abstract

This study aimed to investigate the effect of oxidized beef protein on colon health. C57BL/6 mice were fed diets containing *in vitro* oxidized beef protein (carbonyl content 5.83/9.02 nmol/mg protein) or normal beef protein (control group, carbonyl content 2.27 nmol/mg protein) for 10 weeks. Histological observations showed that oxidized beef protein diet induced notable inflammatory cell infiltrations in colon. The analysis of high-throughput sequencing indicated oxidized beef protein largely altered the composition of gut microbiota (GM) by increasing proinflammatory bacteria (*Desulfovibrio, Bacteroides, Enterorhabdus*) while reducing beneficial bacteria (*Lactobacillus, Akkermansia*). In addition, oxidized beef protein remarkably increased protein fermentation in the colon, which was evidenced by the elevated *i*-butyrate, i-valerate, and ammonia levels in feces. Furthermore, consuming oxidized beef protein destroyed colon barrier functions by decreasing tight junction proteins expression. These changes in colonic ecosystem activated the proinflammatory pathway of lipopolysaccharide/toll-like receptor-4/nuclear factor kappa B (LPS/TLR-4/NF-κB), eventually leading to colonic inflammatory damage in mice. Taken together, these results imply that consuming oxidized beef protein detrimentally regulates GM and impairs colon health.

## Graphical Abstract

Proposed mechanism of dietary oxidized beef protein induces colonic inflammation by altering gut microbiota. 
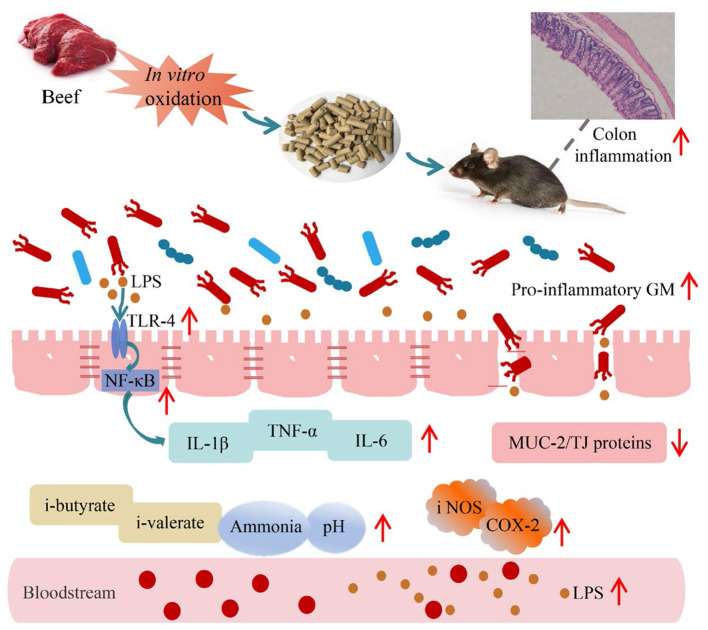

## Introduction

The gut microbiota (GM) is regarded as a hidden organ that plays an important role in regulating host health (1). Numerous chronic diseases such as obesity, diabetes, and colitis are closely linked to GM dysbiosis (2). Some pathogenic bacteria, such as *Desulfovibrio*, and *Helicobacter*, have been confirmed to impair intestinal barrier functions, while some bacteria, such as *Bifidobacterium, Lactobacillus, Akkermansia*, are benefit for intestinal barrier integrity (3). Gut is the first line of defense between host and external environment. Once the function of intestinal barrier is disrupted, the leakage of GM will stimulate immunity systems, leading to inflammation and tissue damage (4). Furthermore, the GM-associated metabolites, e.g., lipopolysaccharides (LPS), flagellins, can bind to toll-like receptors, and then trigger a series of inflammatory responses in the gut (5). Therefore, keeping GM homeostasis is an important target to maintaining gut health.

Diet is a critical factor in regulating GM composition and function (1). As one of the macronutrients, protein is an indispensable component of human diets. After consumption, most proteins are hydrolyzed by digestive enzymes and absorbed in the small intestine, meanwhile, some of the undigested proteins enter colon, where they serve as substrates for GM (4). Proteins can modulate the composition and metabolism of GM by providing nitrogen and carbon (6). There is growing recognition that the composition and metabolic activity of GM can be modulated by the content, processing methods, and source of dietary protein, which in turn impact host health (7). High-protein diets have been reported to increase protein fermentation in colon, altering the composition of GM and exacerbating colitis in mice (8). Wei et al. (9) described that protein from different duck products induced different physiological changes by regulating GM.

Meat and meat products have been parts of the typical western diet. In recent two decades, the influence of consuming processed meat on human health has attracted considerable concern, because a high consumption is suspected to raise the risk of certain cancers progression, such as colorectal cancer (10). Several factors including N-nitroso compounds and lipid peroxidation products have been proposed to explain the probable link between high dietary processed meat and raised colorectal cancer risk (11). However, it is worth noting that protein is the most abundant substance in processed meat products apart from water. Simultaneously, the degree of protein oxidation in processed meat products is substantially higher than in raw meat (12). Intake of oxidized protein has been recognized to increase body oxidative stress and the risk of disease (13). Nevertheless, the impacts of dietary oxidized meat protein on GM community and colon health are still largely unknown.

Here, to investigate the effects of oxidized meat protein on GM composition and colon health, C57BL/6J mice were exposed to diets containing oxidized beef protein for 10 weeks. Colon health was assessed by hematoxylin and eosin staining. In addition, changes in the composition of GM and its associated metabolites, colon barrier functions, and inflammation-related genes were analyzed in the hope of elucidating possible mechanisms. The finding in this study will provide a better understanding about dietary processed meat products on intestinal health.

## Materials and methods

### Preparation of oxidized protein and diets

Semimembranosus muscles obtained from 24-month-old Simmental crossbred beef cattle were purchased from Hengdu Food Co., Ltd. (Zhumadian, China). For protein extraction, beef muscles were minced and filtered with 10 times of volume methanol solvent/methylene chloride (v:v, 1:2) to remove moisture and fat. The filter residue was freeze-dried to obtain beef protein. For different degrees of oxidized beef protein, the above-obtained protein was treated with *in vitro* oxidation systems following a previous study (14). Briefly, beef protein was incubated with oxidizing reagents (5 or 10 mM H_2_O_2_, 10 μM FeCl_3_, and 0.1 mM ascorbic acid) for 24 h at 4°C. After incubation, the mixture was performed dialysis with 3-kDa cut-off dialysis bags [Shanghai Yuanye Biotechnology, Ltd (Shanghai, China)] for 24 h at 4°C, to remove the excess oxidizing reagents. Then, the oxidation-treated protein was further lyophilized for preparing mice feeds. The protein oxidized with 5 mM, and 10 mM H_2_O_2_ was recorded as oxidized beef protein medium lever (OPM) and oxidized beef protein high lever (OPH), respectively. The protein incubated with ultra-pure water instead of oxidizing reagents was used as control and recorded as control protein (CP, low protein high lever).

The mice diets were produced by Trophic Animal Feed High-Tech Co., Ltd. (Nantong, China) following the standard formulation of AIN-93G, with only a change, namely replacing casein with CP, OPM, and OPH, respectively. The diet compositions are presented in Table 1.

**Table 1:** Compositions and analysis of experimental diets.

	**Unit**	**CP diet**	**OPM diet**	**OPH diet**
Diet formulation^a^				
Protein (CP)	g/kg	178.6	0	0
Protein (OPM)	g/kg	0	178.6	0
Protein (OPH)	g/kg	0	0	178.6
Corn starch	g/kg	397.5	397.5	397.5
Dextrinised cornstarch	g/kg	132.0	132.0	132.0
Fiber	g/kg	50	50	50
Sugar	g/kg	100.0	100.0	100.0
Soybean oil	g/kg	29	29	29
Mineral mix	g/kg	35.0	35.0	35.0
Vitamin mix	g/kg	10	10	10
L-Cystine	g/kg	3.0	3.0	3.0
Choline chloride	g/kg	2.5	2.5	2.5
Diet analysis^b^				
Carbonyl content	nmol/mg protein	2.27 ± 0.15c	5.83 ± 0.24b	9.02 ± 0.50a
Sulfhydryl content	nmol/mg protein	57.25 ± 0.52a	39.75± 0.47b	26.29 ± 0.39c
Tryptophan fluoresce intensity	a.u.	10081.0 ± 281.88a	6347.4± 142.67b	2730.8 ± 92.25c
Protein digestibility	%	33.78 ± 0.48a	28.22 ± 0.41b	25.63 ± 0.35c

a The Data of diet formulation was provided by the Trophic Animal Feed High-Tech Company, Ltd. (Nantong, China).

b The Data of diet analysis were expressed in means ± SEM (n = 5). Different letters (a, b, c) in the same line represent differ significantly (p < 0.05).

### Diet analysis

Methods for measurement of carbonyl, sulfhydryl, tryptophan endogenous fluorescence, and protein digestibility, see the Supplementary file.

### Animal and diets

The animal experiment was conducted under the regulations of Ethical Committee of Experimental Animal Center of Nanjing Agricultural University [*SYXK (Su) 2017-0007*]. A total of 24 male, 5-week-old C57BL/6J mice were purchased from Nanjing Biomedical Research Institute (Nanjing, China). The mice were housed in a specific pathogen-free room at a temperature of 25°C, relative humidity of 60 ± 10%, and a 12 h dark/light cycle. After a 7-day acclimation period with standard AIN-93G diet, mice were randomly divided into three groups (8 mice per group). One group received the diet prepared with CP protein (CP diet); the other two groups received the diet prepared with OPM protein (OPM diet) or OPH protein (OPH diet) for 10 weeks. During this time, mice were allowed to eat and drink freely. Food intake and body weight of mice were recorded weekly. Finally, according to humanitarian principles, the mice were anesthetized and sacrificed by dislocating the neck. The serum, colon tissue, colon content, and fresh feces were collected and quickly frozen with liquid nitrogen and then stored in a refrigerator (−80°C) for further analysis.

### Histological evaluation

After fixation with 10% formalin for 72 h, colonic tissues were dehydrated with graded ethanol. The dehydrated tissues were embedded in paraffin. Then, the embedded blocks were cut into 4 μm-thick slices and stained with hematoxylin and eosin. Finally, the slices were further dehydrated with graded ethanol and mounted with neutral gum. The images were viewed with a light microscope.

### Analysis of the microbial community

The composition of GM was determined following a previous study (15). Firstly, the total fecal bacterial DNA was extracted using A Qiagen DNA tool kit (Hilden, Germany). The integrity of isolated DNA was tested by 1% agarose gel electrophoresis; purity and concentration were measured using a ND-1000 spectrophotometer (Thermo, Waltham). The hypervariable regions in V3–V4 of the 16S rRNA gene were amplified by high-fidelity PCR. After that, high-throughput sequencing was performed on the Illumina Miseq platform.

### Analysis of pH, ammonia, and SCFAs contents in feces

The pH measurement was according the method described by Tian et al. (16). Each feces (0.4 g) was mixed with 4 mL neutral water, followed by homogenate using a homogenizer (T25, IKA, Staufen, Germany) at 5,000 rpm for 30 s, and a micro-pH meter was applied to measure pH values. The ammonia content in fecal samples was determined by Berthelot's indophenol reaction (4).

For short-chain fatty acids (SCFAs), each 0.2 g fecal sample was mixed with 1 mL ultra-pure water and homogenized using a homogenizer (IKA). Then, the homogenate was mixed with 1 mL 2-ethylbutyric acid, followed by centrifugation at 10,000 g for 5 min. The obtained supernatant was used for *analysis via a* gas chromatography equipped with a 30 m × 0.25 μm × 0.25 mm DB-FFAP capillary column (Agilent, Wilmington), according to the parameter described by Chen et al. (17).

### Immunofluorescence

Immunofluorescence was performed following a previous study (18). First, sections of colon tissue were treated with EDTA buffer (pH 8.0) and boiled for antigen retrieval. Then, the tissue sections were incubated with the primary antibody (Servicebio, Wuhan, China) for 12 h at 4°C. After that, the slides were washed 3 times with PBS, each time for 5 min. Next, the tissue sections were incubated with the secondary antibody (Servicebio) for 50 min at 25°C in the dark, followed by nuclear staining with 4′,6-diamidino-2-phenylindole. Finally, slides were gently flicked dry and sealed using anti-fluorescence quenching tablets. Images were taken with a fluorescence microscope (Nikon, Tokyo, Japan). The density of claudin-1, occludin, and zonula occluden-1 (ZO-1) was evaluated using Image-pro Plus 6.0 (Media Cybernetics, Silver Spring, USA); the results are expressed in the values relative to those of the CP group.

### Measurement of LPS content in serum

Following the instruction, the LPS contents in serum and colon tissue were measured using ELISA kits (Neobioscience, Shenzhen, China).

### Extraction of RNA and measurement of gene expression

Total RNA was extracted from 20 to 30 mg of colon tissue using RNA extraction kits (Takara, Shanghai, China). The quality of the isolated RNA was assessed using a spectrophotometer (Thermo). Then, the extracted RNA was immediately reversely transcribed to cDNA. After that, the cDNA was mixed with the qPCR master mixture and then analyzed on the real-time PCR system (Quant Studio 6 Flex, Thermo). System parameters were performed as described by Tian et al. (19). Primers used in this work are presented in Supplementary Table S1. Results are expressed in terms of relative gene expression calculated by 2^−ΔΔCt^; GAPDH was set as reference gene.

### Statistical analysis

Results were expressed as mean ± standard error of measurement (SEM) or box plot. Data from GM were analyzed using the R package (V3.4.0). Statistical differences between the CP, OPM, and OPH groups were analyzed by Tukey test using SPSS software 23.0 (IBM Corporation, NY). Significant difference was considered when *p* < 0.05.

## Results

### Characteristics of protein oxidation in the diet

Carbonyl, sulfhydryl, and tryptophan endogenous fluorescence are commonly used indicators to assess protein oxidation. As shown in Table 1, the protein carbonyl in CP diet was 2.27 nmol/mg protein. Compared with CP diet, much higher protein carbonyl was observed in the OPM (5.83 nmol/mg protein) and OPH (9.02 nmol/mg protein) diet, increasing by 1.57- and 2.97-fold, respectively. In addition, Compared to CP diet, there were 30.57 and 54.08% of sulfhydryl lost in the OPM and OPH diet, respectively (*p* < 0.05, Table 1). Moreover, the tryptophan endogenous fluorescence gradual declined from CP to OPH diet (*p* < 0.05, Table 1). Furthermore, protein digestibility decreased by 16.47 and 24.13% in the OPM and OPH diet, compared to CP diet (*p* < 0.05, Table 1). These results suggest that we successfully prepared AIN-93G-like feed that contain different oxidized-level of beef protein.

### Consuming oxidized beef protein reduced body weight and induced colonic damage in mice

**Figure 1:**
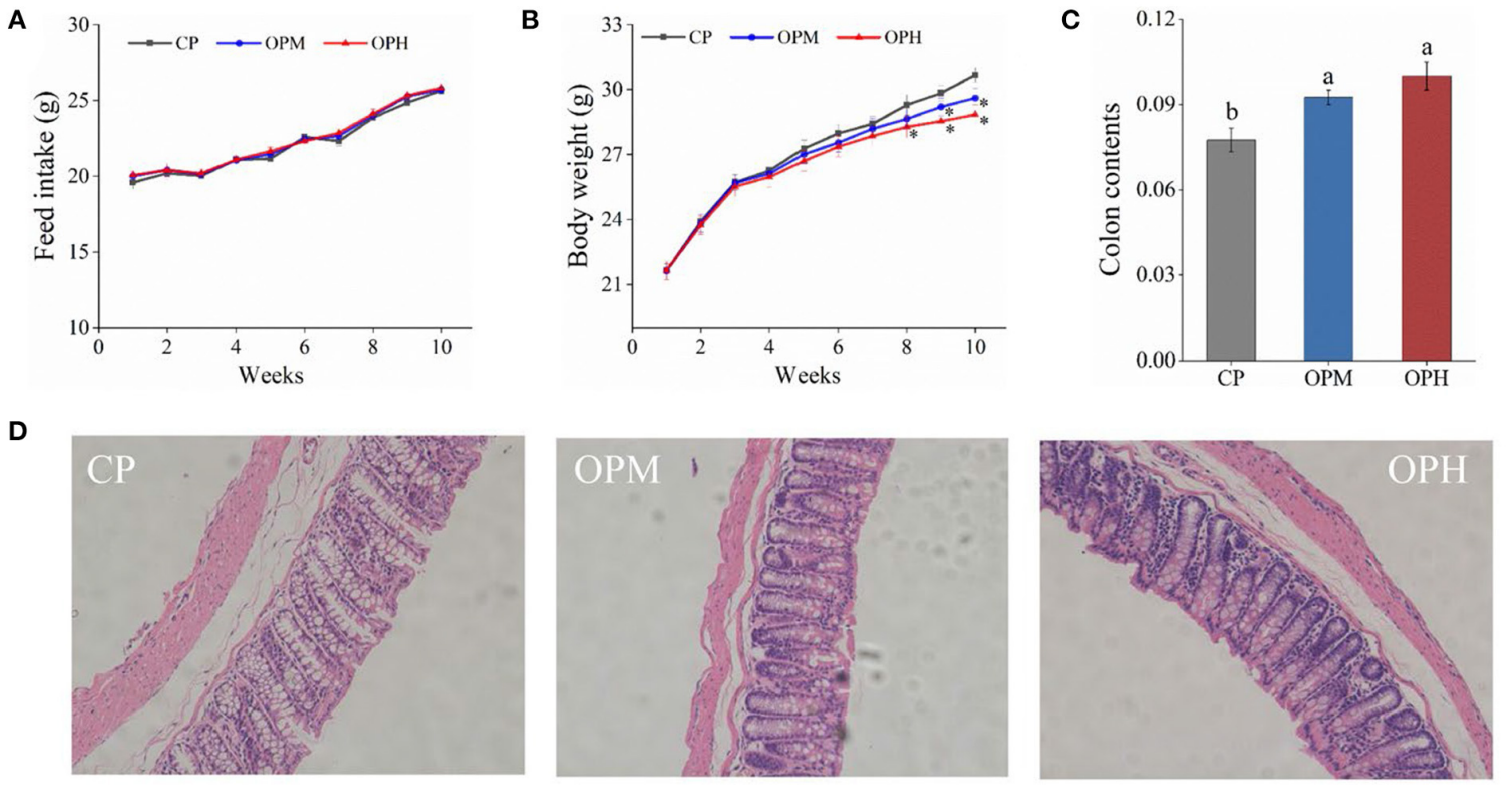
Effects of consuming oxidized beef protein on body weight and colon development. **(A)** Feed intake; **(B)** Body weight; **(C)** Colon contents; **(D)** Colon development. The * indicates a significant difference (*p* < 0.05) from the CP group. Different letters (a,b) represent significant differences (*p* < 0.05). The results were expressed in means ± SEM (*n* = 8).

As shown in Figure 1A, there was no significant difference in feed intake among CP, OPM, and OPH groups (*p* > 0.05). However, compared to CP group, a significant decrease of body weight was observed in the OPH group from 8th to 10th weeks (Figure 1B). At the same time, both OPM and OPH groups had higher colon content than CP group (*p* < 0.05, Figure 1C). The representative images of the histological colon sections are shown in Figure 1D. In CP group, the colon mucosa was intact, with neatly arranged and abundant goblet cells. However, consumption of OPM diet induced colonic damage evidenced by the observation of edema, inflammatory infiltration, and disrupted goblet cell. Such damages were exacerbated in the OPH group. These results indicate that consuming oxidized beef protein causes weight loss, increase of colon contents, and colonic damage in mice.

### Consuming oxidized beef protein altered GM diversity and composition

**Figure 2:**
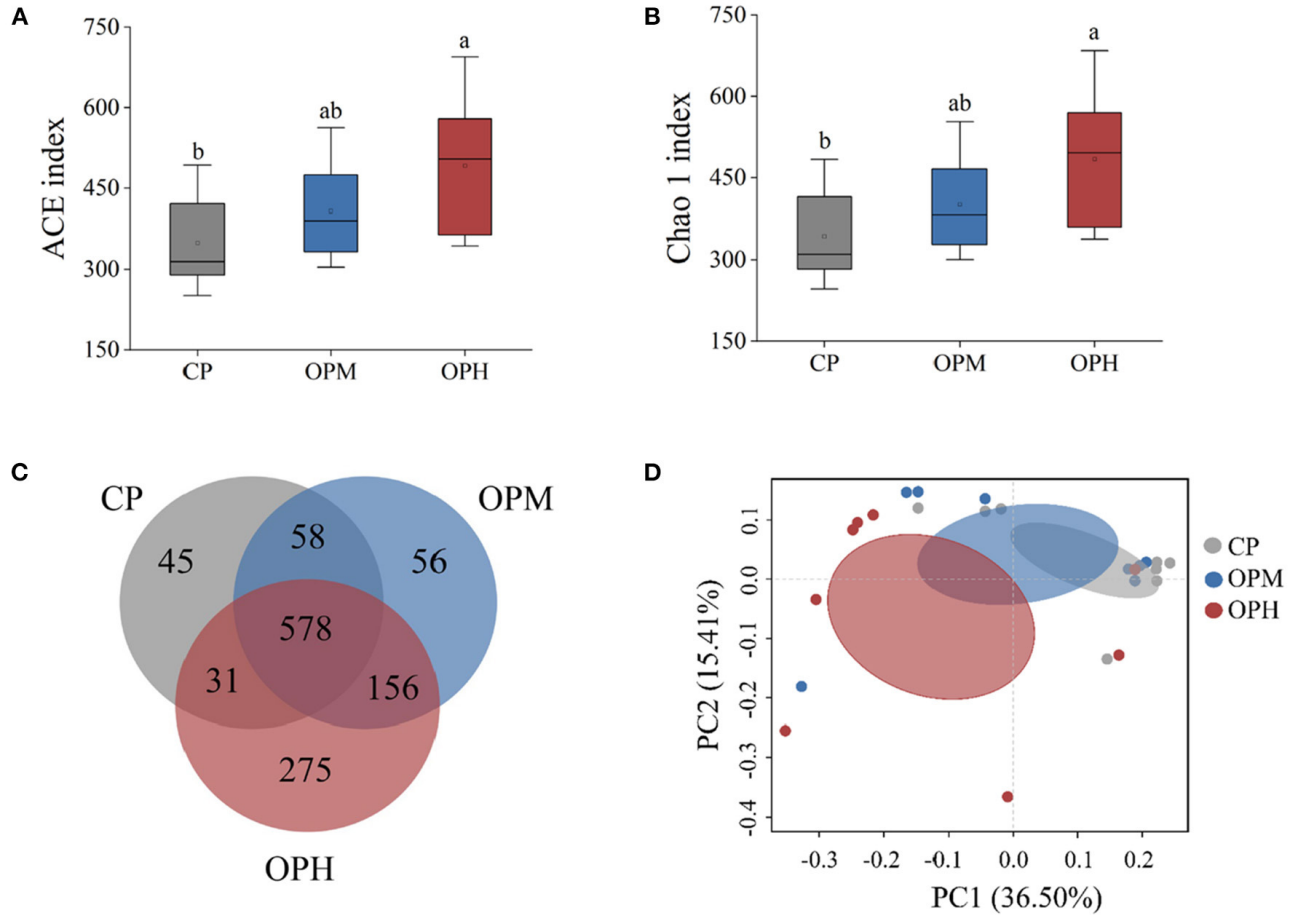
Effects of consuming oxidized beef protein on α and β diversity of GM. **(A)** ACE index; **(B)** Chao1 index; **(C)** PCoA based unweighted unifrac distances; **(D)** Venn diagrams of OTUs. Different letters (a,b) represent significant differences (*p* < 0.05). The results of ACE index and Chao1 index were expressed in box plot with whisker bar (*n* = 8).

To assess changes in GM diversity and richness, the alpha diversity indexes involving ACE, and Chao1 were analyzed. Compared to CP group, significantly increased ACE (Figure 2A), and Chao1 indexes (Figure 2B) were observed in OPH group (*p* < 0.05). No significant difference was found between OPM and OPH groups (Figures 2A,B). To probe into the compositional differences of GM, Venn diagram of OUTs was performed. As shown in Figure 2C, there were 712, 848, and 1,040 OUTs in CP, OPM, and OPH groups, respectively. Among them, there were 45, 56, and 275 unique OUTs in CP, OPM, and OPH. Principal co-ordinates analysis (PCoA) was performed to analyze the beta diversity of GM, which is the common approach to evaluate the similarity/difference of GM community structure among different treatments. As shown in Figure 2D, the three groups of CP, OPM, OPH can be clearly separated by PCoA, where plots in CP group mainly appeared in the upper right corner, while plots in OPH group mainly appeared in the left position. Overall, these results showed that consuming oxidized beef protein significantly increased GM richness and largely altered GM composition.

**Figure 3:**
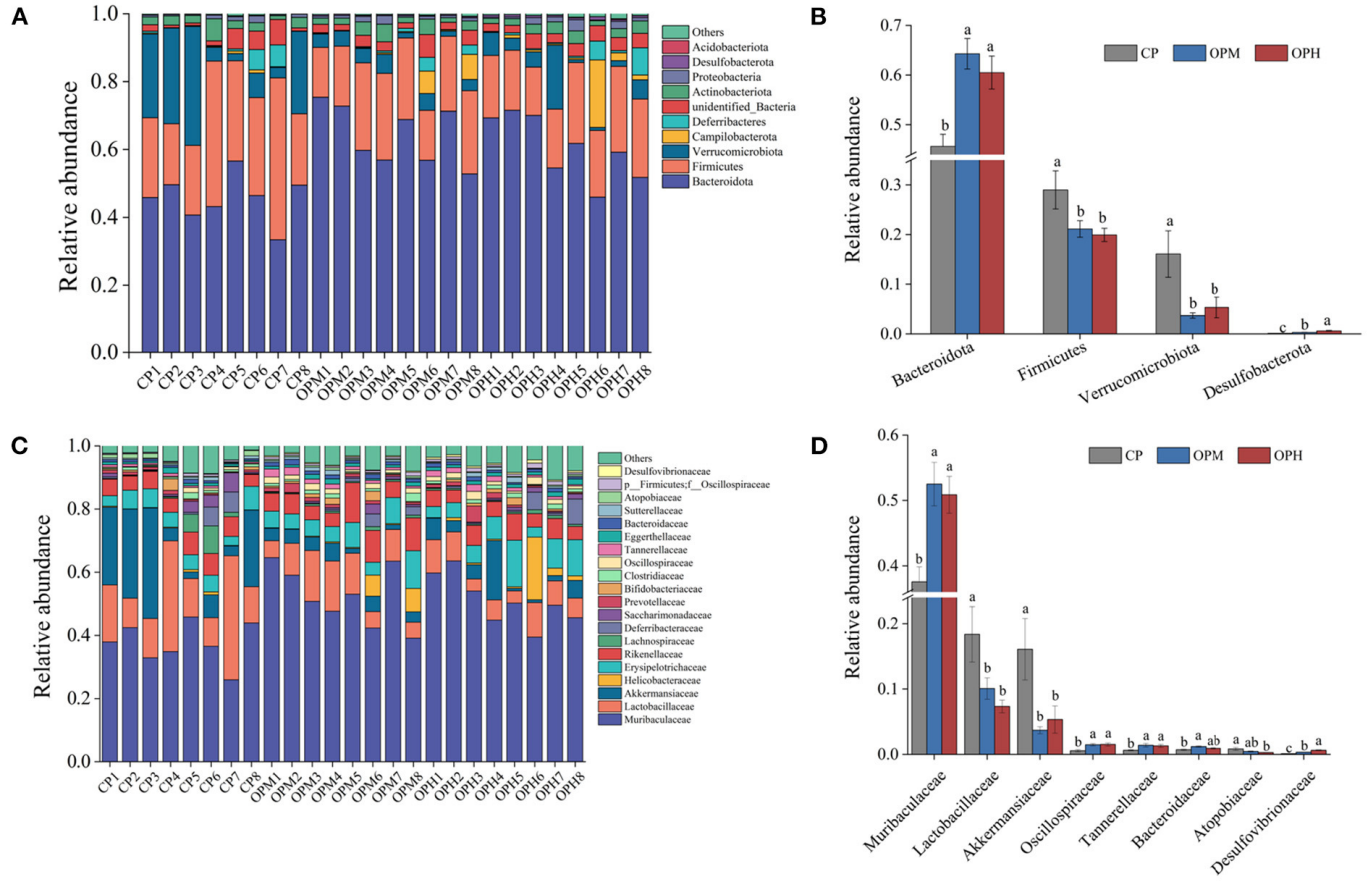
Effects of consuming oxidized beef protein on GM taxonomic profiles. GM composition at phylum level **(A)**, relative abundance at phylum level **(B)**, GM composition at family level **(C)**, relative abundance at family level **(D)**. Different letters (a-c) represent significant differences (*p* < 0.05). The results were expressed in means ± SEM (*n* = 8).

**Figure 4:**
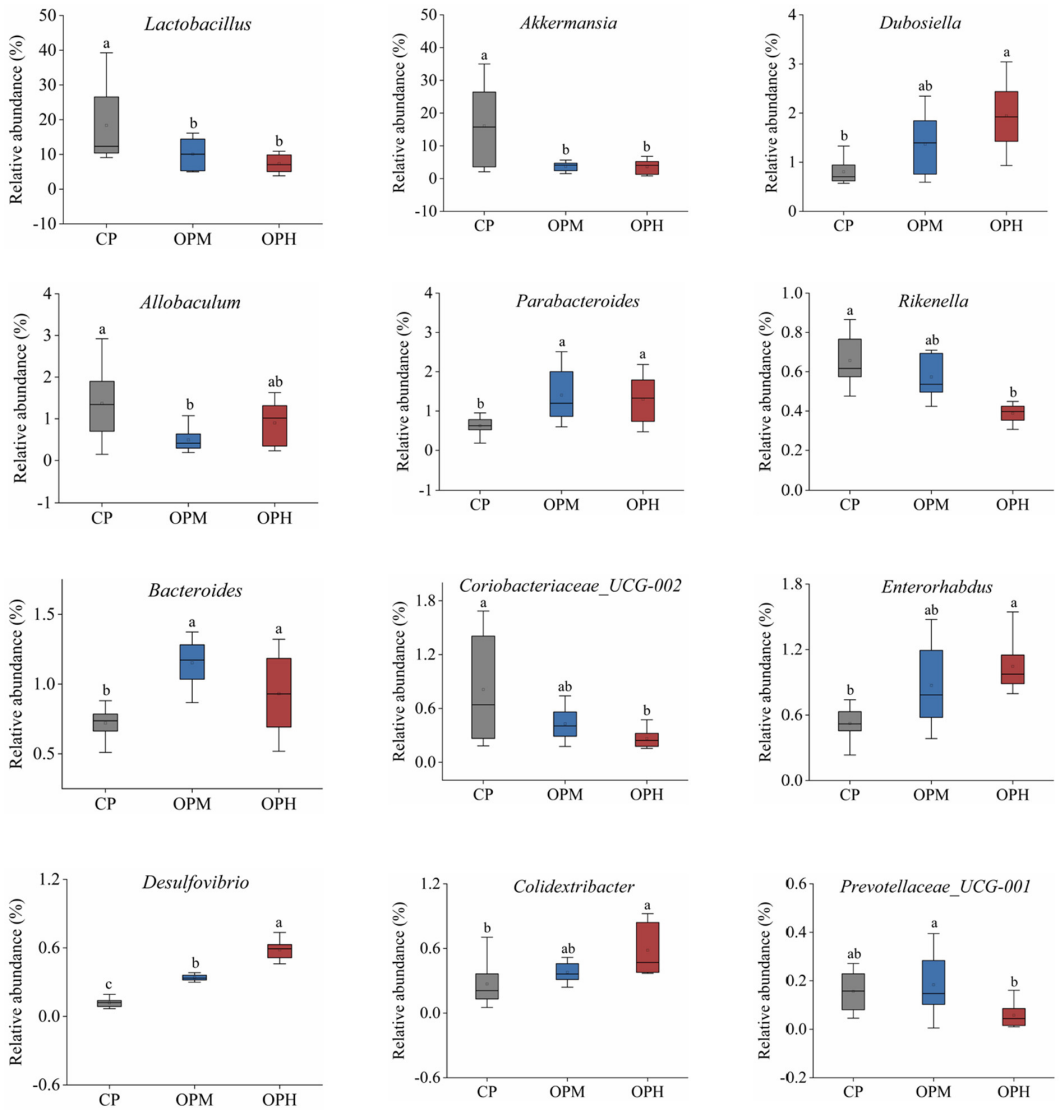
Effects of consuming oxidized beef protein on GM composition at the genus level. Different letters (a-c) represent significant differences (*p* < 0.05). The results of were expressed in box plot with whisker bar (*n* = 8).

To explore the specific variations in the GM composition, the relative abundance of predominant phyla, family, and genus were compared among the three groups. At phylum level, Bacteroidota, and Firmicutes were the major phyla in all the three groups, following by Verrucomicrobiota, Campilobacterota, and Deferribacteres (Figure 3A). Comprised to CP group, the relative abundance of Bacteroidota was markedly elevated (*p* < 0.05), while Firmicutes, and Verrucomicrobiota were remarkably decreased in both OPM and OPH groups (*p* < 0.05, Figure 3B). In addition, consuming oxidized beef protein increased Desulfobacterota in a dose dependent manner (*p* < 0.05, Figure 3B). At family level, Muribaculaceae was the predominant bacterium, which account for 37.54, 52.51, 50.87% in CP, OPM, OPH group, respectively (Figure 3C). The higher relative abundances of Oscillospiraceae, and Tannerellaceae, while lower Lactobacillaceae, and *Akkermansiaceae* were observed in both OPM and OPH groups (*p* < 0.05, Figure 3D). At genus level, changes profile of the top 30 genus is illustrated in Figure 4. Briefly, comprised to CP group, the relative abundances of *Bacteroides, and Parabacteroides* were remarkably enriched, while *Lactobacillus*, and *Akkermansia* were noticeably decreased in both OPM and OPH groups (*p* < 0.05, Figure 4). Meanwhile, the relative abundances of *Dubosiella, Enterorhabdus*, and *Colidextribacter* were significantly increased, while *Coriobacteriaceae_UCG-002*, and *Rikenella* were considerably decreased in the OPH group. In addition, oxidized beef protein enriched *Desulfovibrio* in a dose-dependent manner.

### Consuming oxidized beef protein increased protein fermentation in colon

**Figure 5:**
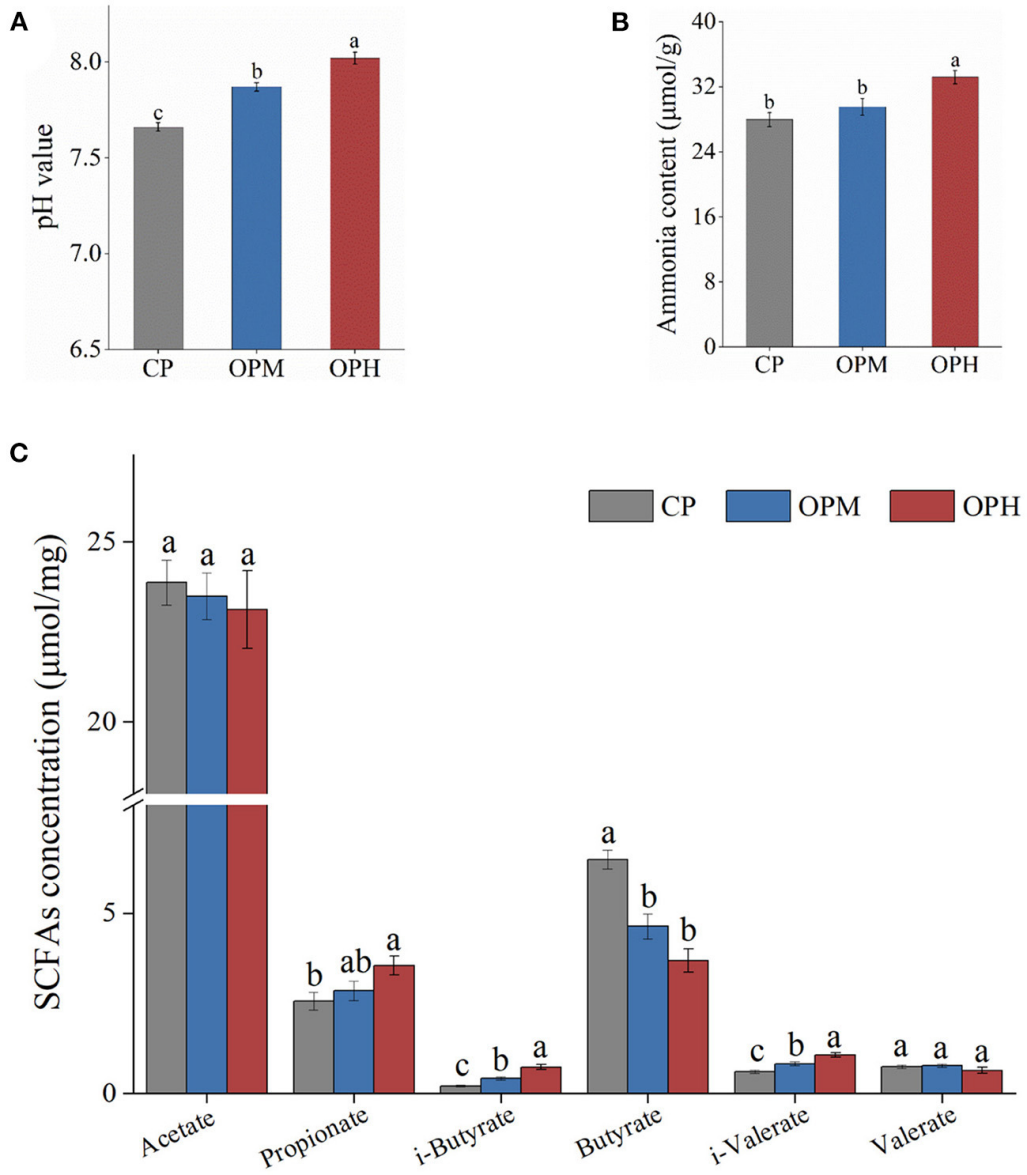
Effects of consuming oxidized beef protein on pH **(A)**, ammonia **(B)**, SCFAs contents **(C)**. Different letters (a-c) represent significant differences (*p* < 0.05). The results were expressed in means ± SEM (*n* = 8).

To evaluate the effect of oxidized beef protein on protein fermentation in colon, pH value, GM related metabolites involving ammonia, SCFAs, in feces were measured. As presented in Figure 5A, the pH in CP group was 7.66. Compared to CP group, consuming oxidized beef protein markedly enhanced fecal pH in a dose-dependent manner (*p* < 0.05). In addition, compared to CP group, the ammonia content was higher in the OPH group, increasing by 18.65% (*p* < 0.05, Figure 5B). The effects of consuming oxidized beef protein on SCFAs were mainly reflected in branched chain fatty acids. As shown in Figure 5C, consuming oxidized beef protein increased both i-butyrate and i-valerate contents in a dose-dependent manner. Besides, compared to CP group, the propionate content was significantly increased, while the butyrate content was markedly reduced in OPH group (*p* < 0.05, Figure 5C). No difference was observed in acetate, and valerate among the three groups (*p* > 0.05). The i-butyrate and i-valerate are hallmarks of GM-fermented proteins. Therefore, the above results indicate consuming oxidized beef protein increased protein fermentation in the colon, and altered colonic microenvironment with increased pH, ammonia, and propionate, while decreased butyrate.

### Consuming oxidized beef protein impaired colon barrier functions

**Figure 6:**
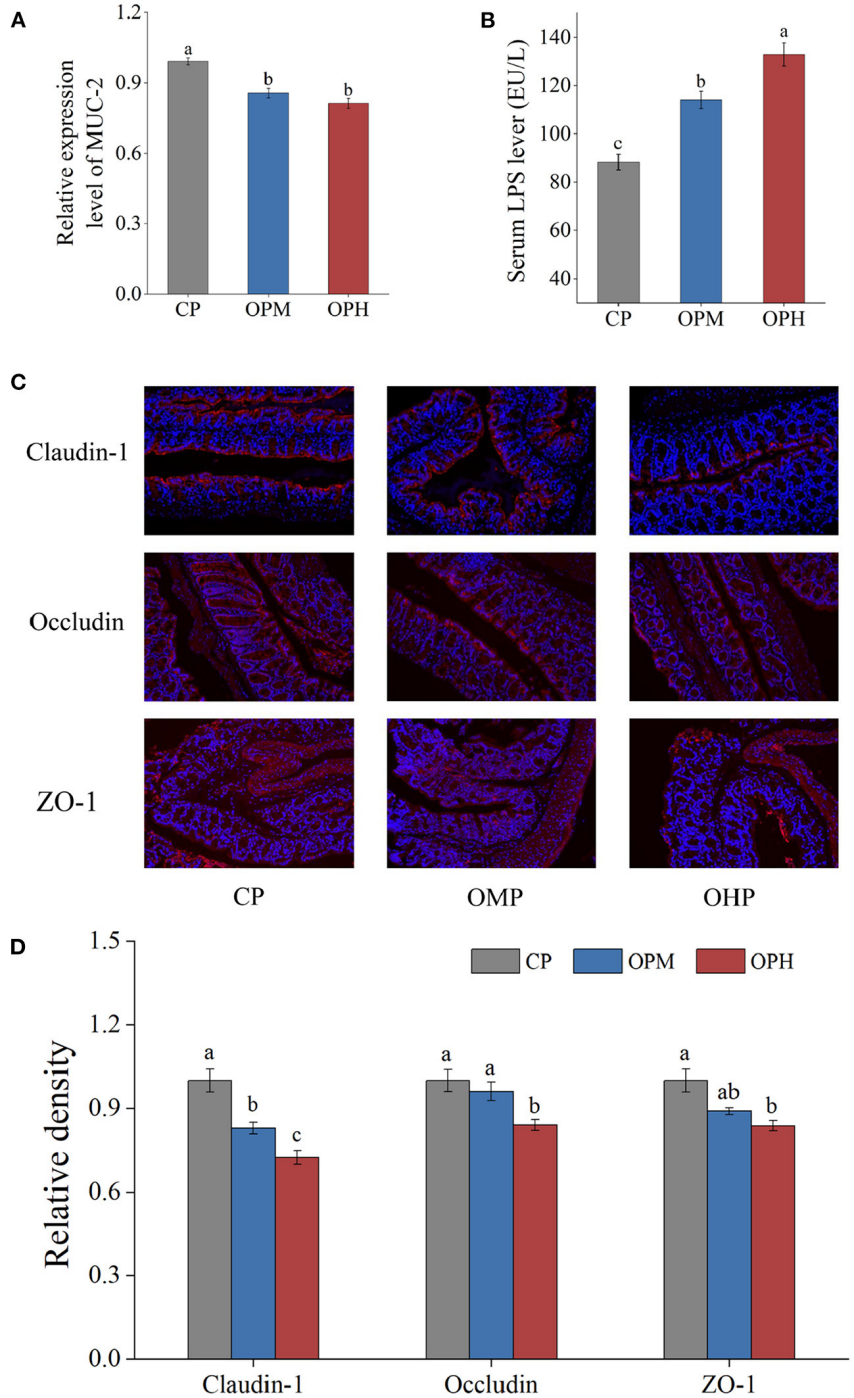
Effects of consuming oxidized beef protein on colonic barrier function. The mRNA expression of MUC-2 **(A)**, LPS content in serum **(B)**, representative images of immunofluorescence of claudin-1, occludin, and ZO-1, original magnifications: × 200 **(C)**, the relative density of claudin-1, occludin, and ZO-1 **(D)**. Different letters (a-c) represent significant differences (*p* < 0.05). The results were expressed in means ± SEM (*n* = 8).

The mucus layer, primarily formed by mucin-2 (MUC-2), is the first line of colonic barrier. As presented in Figure 6A, comprised to CP group, the mRNA expressions of MUC-2 were remarkably decreased in both OPM and OPH groups. LPS is a marker of intestinal permeability. To further determined the influence of consuming oxidized beef protein on colonic barrier, the LPS level in serum was measured. As showed in Figure 6B, consuming oxidized beef protein markedly increased LPS content in serum in a dose-dependent manner. Tight junction proteins involving claudin-1, occludin, and ZO-1 are commonly used to evaluate colonic barrier integrity. To better assess whether dietary oxidized beef protein cause colonic barrier damage, the immunofluorescence measurements of claudin-1, occludin, ZO-1 were performed. The representative images are shown in Figure 6C, where claudin-1, occludin, and ZO-1 were stained red, while the nuclei were stained blue. Compared to CP group, the number of red spots representing claudin-1 apparently decreased in both OPM and OPH groups in a dose-dependent manner (*p* < 0.05, Figures 6C,D). Simultaneously, the expressions of occludin and ZO-1 were considerably decreased in the OPH group (*p* < 0.05, Figures 6C,D). Taken together, these results suggest that dietary oxidized beef protein impairs colonic barrier by reducing the expressions of MUC-2, claudin-1, occludin, and ZO-1, which increased colonic permeability that led to elevation of LPS in serum.

### Consuming oxidized beef protein activated LPS/TLR-4/NF-κB proinflammatory pathway in colon

**Figure 7:**
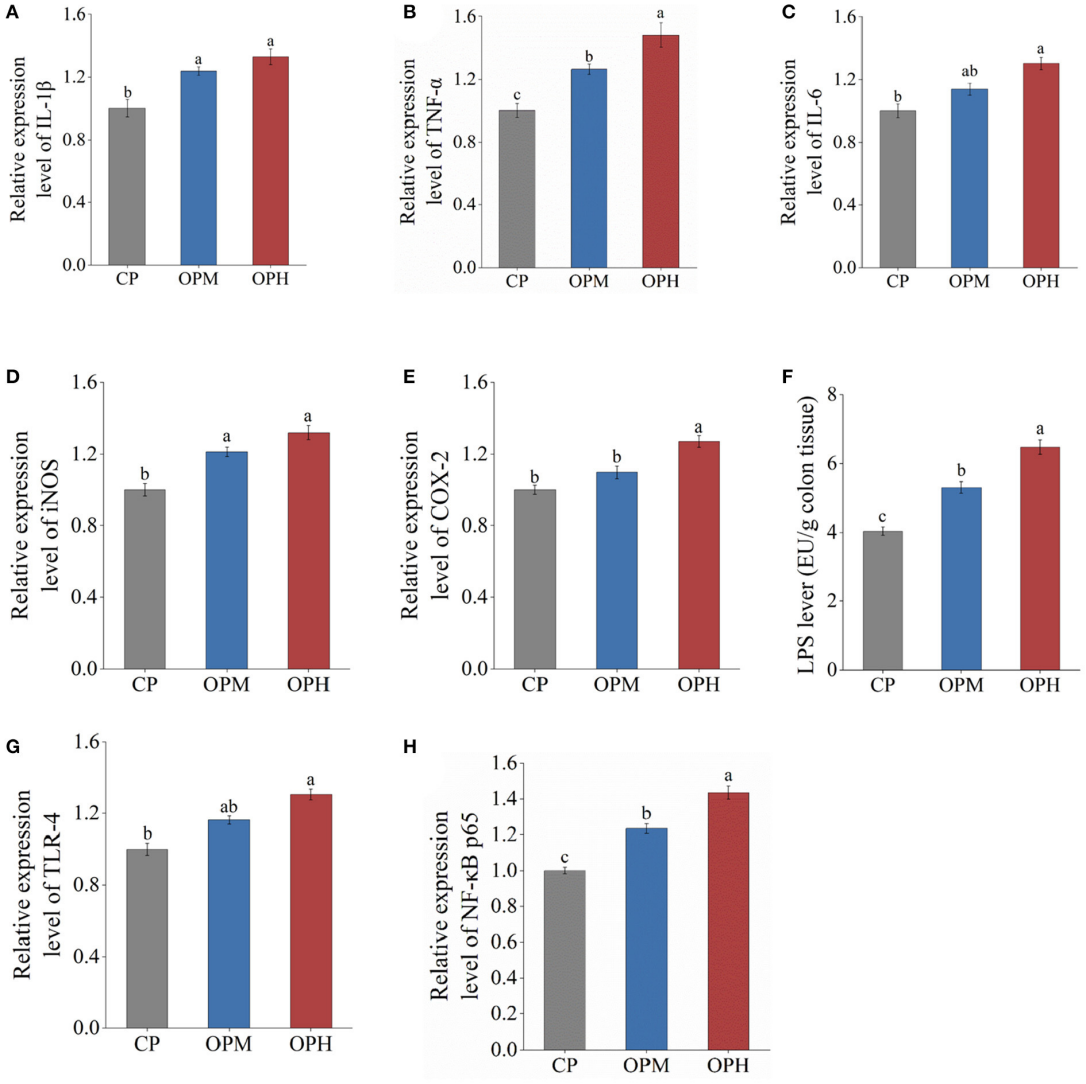
Effects of consuming oxidized beef protein on proinflammatory factors. **(A)** IL-1β; **(B)** TNF-α; **(C)** IL-6; **(D)** iNOS; **(E)** COX-2; **(F)** LPS; **(G)** TLR-4; **(H)** NF-κB p65. Different letters (a-c) represent significant differences (*p* < 0.05). The results were expressed in means ± SEM (*n* = 8).

The inflammatory response is closely related to the over-expressions of proinflammatory cytokines involving interleukin-1β (IL-1β), tumor necrosis factor-α (TNF-α), and interleukin-6 (IL-6). As shown in Figure 7A, compared to CP group, the expressions of IL-1β were significantly upregulated in both OPM and OPH groups (*p* < 0.5). Besides, the expression of TNF-α remarkably increased from OP group to OPH group (*p* < 0.5, Figure 7B). In addition, compared to CP group, the expression of IL-6 increased by 30.37% in OPH group (*p* < 0.5, Figure 7C). The enzymes of inducible nitric oxide synthase (iNOS) and cyclooxygenase 2 (COX-2) are pivotal to the assessment of inflammation levels, which triggers nitric oxide and reactive oxygen species production, respectively. The expression of iNOS was markedly increased in both OPM and OPH groups (*p* < 0.5, Figure 7D). Simultaneously, the expression of COX-2 was higher in OPH group than CP group (*p* < 0.5, Figure 7E).

Based on the enhanced LPS in serum, impaired intestinal barrier, and altered GM composition, the potential molecular mechanisms underlying oxidized beef protein- induced inflammation were explored from the LPS/TLR-4/NF-κB pathway. The result showed that dietary oxidized beef protein remarkably increased LPS content in the colon tissue (*p* < 0.5, Figure 7F). Simultaneously, consuming oxidized beef protein markedly upregulated the mRNA expressions of TLR-4, and NF-κB in a dose-dependent manner (*p* < 0.5, Figures 7G,H). Taken together, these results suggest that consuming oxidized beef protein activated the proinflammatory signaling pathway of LPS/TLR-4/NF-κB in colon.

## Discussion

Meat and meat products are widely consumed by consumers for the enrichment of all essential amino acids, micro-nutrients, and tempting sensory attributes (10). However, meat protein is susceptible to oxidation during processing, which increases the potential risk of dietary oxidized protein for humans (13). To evaluate the impact of oxidized meat proteins, meanwhile minimize other confounding factors in feed, e.g., fat oxidation products, AGEs. In this work, we first prepared different levels of oxidized beef proteins *in vitro* oxidation system, and then apply them instead of casein to prepare AIN-93G like feed. As expected, the protein oxidation level in CP, OPM, and OPH diet gradually increased. Digestibility is an essential indicator for assessing the nutritional value of dietary protein. The reduction in protein digestibility not only decreases amino acid bioavailability, but also increases protein fermentation in the colon that may impair host health (4, 6). The decreased protein digestibility observed in OPM, and OPH diets could be explained by the fact that protein carbonylation increased resistances to digestive enzymes (20).

Growing evidence shows that dietary proteins can modulate GM and consequently affect gut health (6, 7). In this study, we observed that consumption of oxidized beef protein markedly enhanced GM richness. This phenomenon could be due to that protein oxidation led to a decrease in protein digestibility, allowing more undigested protein to enter colon, which favored the growth of GM (21). In addition, the GM richness is associated with colonic transit time (22). The increase of colon content driven by oxidized beef protein prolonged colonic transit time, thus also leading to the elevation of GM richness. The increased GM richness is mainly attributed to the enrichment of Bacteroidota, while reduction in Firmicutes (Figures 3A,B). Bacteroidota is known as the dominant proteolytic bacterium with broad abilities to secrete proteases and peptidases (23). Therefore, it is not surprising that consuming oxidized beef protein enhanced the abundance of Bacteroidota, while decreased Firmicutes that preferentially ferment carbohydrates (24). Both ulcerative colitis and inflammatory bowel disease patients are charactered by increase of Bacteroidota, meanwhile, decrease of Firmicutes (3, 8). Thus, the changes in phylum-level of GM driven by oxidized beef protein may be detrimental to colon health.

At genus level, oxidized beef protein remarkably enriched the abundances of *Desulfovibrio, Bacteroides, Enterorhabdus*, and *Colidextribacter*, while reduced *Lactobacillus, Akkermansia*, and *Rikenella*. *Desulfovibrio* is a typical proinflammatory bacterium, which produces the harmful metabolite of H_2_S (7). The increase of *Desulfovibrio* driven by oxidized beef protein probably related to oxidation of sulfur-containing amino acids. Fu et al. (25) pointed out that sulfur-containing amino acids in beef protein, e.g., cysteine and methionine, are susceptible to oxidation. Moreover, it has demonstrated that the oxidation derivatives of cysteine (cysteine-sulfinic acid), and methionine (methionine sulfone) cannot be absorbed by the small intestine (26). Thus, consuming oxidized beef protein might deliver more oxidation derivatives of sulfur-containing amino acids to the colon, thereby promoting the growth of *Desulfovibrio*. *Bacteroides* is the dominant proteolytic genus, whose enhancement driven by oxidized beef protein could be associated with the accumulation of protein in colon due to the reduction of protein digestibility (23). Although *Bacteroides* are sometimes related to leanness and host health, some of their species, such as *Bacteroides fragilis, Bacteroides vulgatus*, are confirmed to induce inflammation and metabolic disease (27). *Enterorhabdus* belongs to the Coriobacteriaceae family and *Coriobacteriia* class, which is associated with ileocecal mucosa inflammation (28). A recent study reported that a low-tryptophan diet greatly increased the abundance of *Enterorhabdus*, leading to systemic inflammation in mice (29). *Colidextribacter* is correlated with hyperlipidemia and oxidative stress (30). Esteves et al. (31) reported that the relative abundance of *Colidextribacter* increased ~6-fold in colitis mice, compared to normal mice.

On the contrary, the reduced *Lactobacillus and Akkermansia* driven by oxidized beef protein are well-known probiotics. It is well-documented that *Lactobacillus* can be supplied as an adjuvant for preventing colonic inflammation by regulating host immunity (32). Besides, *Lactobacillus* is able to inhibit the growth of pathogenic bacteria and improve disease *via* producing lactic acid, antimicrobial peptides, and hydrogen peroxide (33). A recent study reported that *Lactobacillus gallinarum*-produced indole-3 lactic acid remarkably reduced the proliferation of colon cancer tumors (34). *Akkermansia* is the main LPS-suppressing genus that contributes to intestinal health (35). Reduction of *Akkermansia* is commonly observed in mice with colitis (3). Conversely, oral administration of *Akkermansia* considerably ameliorates intestinal inflammation, and increases intestinal barrier integrity (35, 36). The decline of *Akkermansia* driven by oxidized beef protein might due to the decrease of mucus layer (Figure 6A). As is known to us, *Akkermansia* is mainly colonized in the intestinal mucins that are formed with highly glycosylated proteins, which is mainly composed with serine and threonine (37, 38). However, serine and threonine are susceptible to be oxidized to hydroxyserine, and hydroxythreonine (39), which might reduce their availability for the formation of mucins, leading to the decrease of ecological niche for *Akkermansia*. Our results are in line with Ge et al. (21), who reported that consumption of pork cooked at high temperature (higher protein oxidation level) reduced the abundances of *Lactobacillus* and *Akkermansia*, compared with consuming pork cooked at low temperature. *Rikenella* belongs to Rikenellaceae family that can ferment SCFAs to provide energy for colon cells (40). Inflammatory bowel disease has reported to linked with decrease in *Rikenellaceae* (35). Overall, the above results indicated that consuming oxidized beef protein altered GM composition in a manner by increasing proinflammatory bacteria (*Desulfovibrio, Bacteroides, Enterorhabdus*), while reducing beneficial bacteria (*Lactobacillus, Akkermansia, Rikenella*).

Apart from GM itself, the GM-related metabolites also performed a critical tie between diet and host health (6). In present study, consuming oxidized beef protein significantly increased protein in colon as evidenced by the rising i-butyrate and i-valerate levels in feces. Generally, excessive protein fermentation is detrimental to colon health due to the production of toxic substances such as ammonia, indoles, H_2_S, and phenols (7). Ammonia has been reported to decrease the renewal of intestinal epithelial cells and impair intestinal barrier functions (41). SCFAs are products of GM metabolisms from both carbohydrates and amino acids (6). In line with our result, higher propionate content was recorded in the cooked meat (higher protein oxidation) than that of raw meat during *in vitro* fermentation (42). Butyrate has been proved to has an anti-inflammatory effect and benefit for maintaining intestinal integrity by activating G-protein-coupled receptors (16). The decrease of butyrate driven by oxidized beef protein might be related to the decline of carbohydrate-fermenting bacteria in *Firmicutes* (Figure 3B). In addition, these metabolites altered the intestinal microenvironment with increased pH. High pH is generally detrimental to intestinal health because it encourages the proliferation of pathogenic bacteria while inhibits the growth of beneficial bacteria (43). Besides, alkaline pH promoted the activity of proteases, which in turn further facilitated protein fermentation in colon (41).

The intestinal barrier allows nutrient absorption while prevents the translocation of microorganisms and their products. Under normal conditions, the intact intestinal barrier can prevent LPS from moving to systemic circulation (44). In present study, consuming oxidized beef protein increased colonic permeability with downregulation of MUC2, claudin-1, occludin, and ZO-1, which led to the leakage of LPS. In line with our results, a previous study reported that dietary oxidized milk protein impaired ileum integrity in mice (45). One explanation for this phenomenon is that consuming oxidized beef protein increased oxidative stress in the colon tissue, which impaired epithelial cell viability (13, 46). In addition, accumulating evidence shows that GM dysbiosis can lead to intestinal barrier dysfunction. For example, *Desulfovibrio* produces H_2_S that decreases disulfide bonds in the mucus network, leading to the impairment of mucus layer (47). In contrast, *Akkermansia* is able to maintain intestinal integrity by promoting the differentiations of Paneth cell and goblet cell (44). In addition, *Akkermansia*-derived extracellular vesicles have been reported to upregulate occludin that favor intestinal barrier integrity (48). On the other hand, as mentioned above, the alterations of GM-related metabolites and intestinal microenvironment driven by oxidized beef protein might also induce the destruction of colon barrier (6).

The colonic inflammatory status is closely linked to its innate immune responses (3). Numbers studies have reported the GM dysbiosis that induced by unhealthy diets, such high fat diet, triggers the LPS/TLR-4/NF-κB proinflammatory pathway and leads to intestinal inflammatory damage (5, 16). As mentioned above the dysfunction of colonic barrier leads to a rising of circulating endotoxins. The endotoxin of LPS is mainly derived from the cell wall of Gram-negative bacteria; TLR-4 is a canonical receptor for LPS and acts as an important interface between GM and host immunity (49). Importantly, the interactions between LPS and TLR-4 will trigger the activation of NF-κB (5). Generally, the NF-κB is exists as an inactive trimer in cytoplasm. Once activated, the subunit (NF-κB p65) enters nucleus and then stimulates the release of inflammatory factors from immune cells, triggering a subsequent series of inflammatory responses (50). Based on the significantly increased LPS, remarkable upregulation of TLR-4 and NF-κB p65, it can be speculated that consuming oxidized beef protein activated the LPS/TLR-4/NF-κB pathway, ultimately leading to colonic inflammatory damage.

In summary, the present study reveals that oxidized beef protein induces colonic inflammatory damage in mice, which is closely related to GM dysbiosis. Consuming oxidized beef protein largely altered GM structure and composition, with an enrichment of proinflammatory bacteria (*Desulfovibrio, Bacteroides, Enterorhabdus*, and *Colidextribacter*) and a reduction of beneficial bacteria (*Akkermansia, Lactobacillus, and Rikenella*). Additionally, consuming oxidized beef protein increased protein fermentation in the colon and impaired colon barrier functions. These events driven by oxidized beef protein triggered the proinflammatory pathway of LPS/TLR-4/NF-κB, which promoted the release of inflammatory factors (IL-1β, IL-6, TNF-α, iNOS, and COX-2) and consequently led to colonic inflammatory damage.

## Data availability statement

The datasets presented in this study can be found in the SRA database (https://www.ncbi.nlm.nih.gov) under accession number: PRJNA872483.

## Ethics statement

The animal study was reviewed and approved by Ethical Committee of Experimental Animal Center of Nanjing Agricultural University [SYXK (Su) 2017-0007].

## Author contributions

YY: conceptualization, methodology, data curation, and writing-original draft. JC: resources and data curation. LZ: resources and software. LX: methodology and writing-original draft. WZ: writing-review and editing, supervision, and funding acquisition. All authors contributed to the article and approved the submitted version.

## Funding

This work was supported by China Agriculture Research System of MOF and MARA (CARS-35), and the earmarked fund for Jiangsu Agricultural Industry Technology System [JATS (2020)425].

## Conflict of interest

The authors declare that the research was conducted in the absence of any commercial or financial relationships that could be construed as a potential conflict of interest.

## Publisher's note

All claims expressed in this article are solely those of the authors and do not necessarily represent those of their affiliated organizations, or those of the publisher, the editors and the reviewers. Any product that may be evaluated in this article, or claim that may be made by its manufacturer, is not guaranteed or endorsed by the publisher.

## Abbreviations

GM: gut microbiota
LPS: lipopolysaccharides
TLR-4: toll-like receptor-4
NF-κB: nuclear factor kappa B
OPM: oxidized beef protein medium lever
OPH: oxidized beef protein high lever
CP: control protein
SCFAs: short-chain fatty acids
SEM: standard error of measurement
PCoA: principal co-ordinates analysis
MUC-2: mucin-2
IL-1β: interleukin-1β
TNF-α: tumor necrosis factor-α
IL-6: interleukin-6
iNOS: inducible nitric oxide synthase
COX-2: cyclooxygenase 2.
