# Supplementary material for: Dietary oxidized beef protein alters gut microbiota and induces colonic inflammatory damage in C57BL/6 mice
Source: Front Nutr. 2022 Sep 2;9:980204. doi: 10.3389/fnut.2022.980204 (PMC9478438; doi:10.3389/fnut.2022.980204)
Supplement: Supplementary file 1 [file Data_Sheet_1.docx]

**Dietary Oxidized Beef Protein Alters Gut Microbiota and Induces** **Colonic Inflammatory Damage in C57BL/6 Mice**

Yantao Yin, Jiaming Cai, Lei Zhou, Lujuan Xing, Wangang Zhang^#^

*Key Laboratory of Meat Processing and Quality Control, Ministry of Education China, College of Food Science and Technology, Nanjing Agricultural University, Nanjing 210095, China*

Corresponding author: Wangang Zhang^#^ ([wangang.zhang@njau.edu.cn](mailto:wangang.zhang@njau.edu.cn))

**Supporting Information**

1. **Measurement of carbonyl content**

The determination of carbonyl following the method described by Zhang et al [1]. Each of 2 g feed was homogenized in 20 mL of pyrophosphate butter (2.0 mM Na_4_P_2_O_7_, 10 mM trizma-maleate, 100 mM KCl, 2.0 mM MgCl_2_, and 2.0 mM EGTA, pH 7.4) using a homogenizer (IKA, Staufen, Germany). Then, 2mL of the homogenate was mixed with equal volume of trichloroacetic acid (TCA, 20%) followed by centrifugation. After centrifugation, the precipitant was incubated with 2 mL of 10 mM DNPH solution (dissolved in 2 M HCl) at 37 °C for 1 h. Then, the mixture was further precipitated with 2 mL of TCA (20%) and centrifuged at 12,000 for 10 min. After that, the precipitate was washed 4 times with 2 mL of ethanol and ethyl acetate (1:1, v/v). Then, the pellet was dissolved in 2 mL of 6 M guanidine solution and centrifuged for 10 min at 5,000 g. The sample incubated with 2 M HCl instead of the DNPH solution served as blank. The carbonyl content was calculated with the absorption coefficient (22,000 M^−1^ cm^−1^).

1. **Measurement of sulfhydryl content**

The determination of sulfhydryl following the method described by Kang et al. [2]. Firstly, 2 g feed was homogenized in 10 mL of butter (0.6 M NaCl, 20 mM PBS). Then, 0.5 mL homogenized sample (1 mg/mL protein concentration) was mixed with 5 mL buffer solution (10 mM ethylenediaminetetraacetic acid, 8 M urea, 20 mM Tris-HCl, pH 6.0) and 100 μL of 10 mM 5,5-dithio-bis (2-nitrobenzoic acid). The mixture was incubated at room temperature for 0.5 h. The absorbance was read using a spectrophotometer (U-3900, Hitachi Corp., Tokyo, Japan) at 412 nm. The SH content was calculated using a molar extinction coefficient of 13,600 M^-1^ cm^-1^. The result was expressed as nmol/mg protein.

1. **Measurement of tryptophan endogenous fluorescence**

The determination of tryptophan endogenous fluorescence following the method described by Zhao et al. [3]. The emission spectra of tryptophan were recorded from 300 to 400 nm at 283 nm excitation wavelengths with sample solutions (0.5 mg/mL). The result was expressed as the maximum fluorescence intensity value.

1. **Measurement of protein digestibility**

The gastric fluid (SGF) and the simulated intestinal fluid (SIF) were prepared according to the standard described by Minekus et al. [4]. For gastric digestion, each of 1 g feed was redissolved in 4 mL of SGF. Then, pepsin was added to the mixture to achieve an enzymatic activity of 2,000 U/mL for initiating gastric digestion. The mixture was incubated in a shaker at 37 °C at 150 rpm. After 120 min of gastric digestion, 1 mL of digested chyme was taken out and immediately mixed with the same volume of SIF to inactivate pepsin. For intestinal digestion, 3 mL of digested chymes were mixed with 3 mL of SIF, and then α-chymosin and trypsin were added to achieve final enzymatic activities of 25 U/mL and 100 U/mL, respectively. The mixture was also reacted in a shaker at 37 °C under 150 rpm for 2 h. Then, the gastrointestinal digestion chyme was heated at 95 °C for 5 min to inactivate α-chymosin and trypsin. Protein digestibility was determined by the degree hydrolysis (DH) of protein following our previous work [5].

**Table S1. Primer Sequences Used for qRT-PCR Analysis**

| Primer | Forward primer | Reverse primer |
| --- | --- | --- |
| MUC-2 | GCTGACGAGTGGTTGGTGAATG | GATGAGGTGGCAGACAGGAGAC |
| Claudin-1 | AGCTGCCTGTTCCATGTACT | CTCCCATTTGTCTGCTGCTC |
| Occludin | ACGGACCCTGACCACTATGA | TCAGCAGCAGCCATGTACTC |
| ZO-1 | ACCCGAAACTGATGCTGTGGATAG | AAATGGCCGGGCAGAACTTGTGTA |
| IL-1β | ACTCATTGTGGCTGTGGAGA | TTGTTCATCTCGGAGCCTGT |
| TNF-α | CCCTCACACTCAGATCATCTTCT | CTACGACGTGGGCTACAG |
| IL-6 | CTCTGGCGGAGCTATTGAGA | AAGTCTCCTGCGTGGAGAAA |
| iNOS | GGGCTGACCTGTTTCCTACT | GGAGGTTGAGACCCAATGGA |
| COX-2 | CCCATTAGCAGCCAGTTGTC | CAGGATGCAGTGCTGAGTTC |
| TLR-4 | AGTGCCCCGCTTTCACCTCT | TCCGGCTCTTGTGGAAGCCT |
| NF-κB p65 | ACGATCTGTTTCCCCTCATCT | TGCTTCTCTCCCCAGGAATA |
| GAPDH | TGGAGAAACCTGCCAAGTATGA | TGGAAGAATGGGAGTTGCTGT |

**References**

1. Zhang, W., Xiao, S., Lee, E. J., & Ahn, D. U. (2011). Consumption of oxidized oil increases oxidative stress in broilers and affects the quality of breast meat. Journal of Agricultural and Food Chemistry, 59 (3), 969-974.
2. Kang, D., Zou, Y., Cheng, Y., Xing, L., Zhou, G., & Zhang, W. (2016). Effects of power ultrasound on oxidation and structure of beef proteins during curing processing. Ultrasonics Sonochemistry, 33, 47-53.
3. Zhao, X., Xing, T., Wang, P., Xu, X., & Zhou, G. (2019). Oxidative stability of isoelectric solubilization/precipitation-isolated PSE-like chicken protein. Food Chemistry, 283, 646-655.
4. Minekus, M., Alminger, M., Alvito, P., Ballance, S., Bohn, T., Bourlieu, C., Carrière, F., Boutrou, R., Corredig, M., & Dupont, D. (2014). A standardised static in vitro digestion method suitable for food–an international consensus. Food & Function, 5 (6), 1113-1124.
5. Yin, Y., Zhou, L., Pereira, J., Zhang, J., & Zhang, W. (2020). Insights into digestibility and peptide profiling of beef muscle proteins with different cooking methods. Journal of Agricultural and Food Chemistry, 68 (48), 14243-14251.
